# Plant Manipulation by Gall-Forming Social Aphids for Waste Management

**DOI:** 10.3389/fpls.2019.00933

**Published:** 2019-07-23

**Authors:** Mayako Kutsukake, Keigo Uematsu, Takema Fukatsu

**Affiliations:** ^1^Bioproduction Research Institute, National Institute of Advanced Industrial Science and Technology (AIST), Tsukuba, Japan; ^2^Department of General Systems Studies, University of Tokyo, Tokyo, Japan

**Keywords:** social aphid, gall, manipulation, waste management, plant cuticle, trichome

## Abstract

Many social aphids form spectacular galls on their host plants, in which hundreds to thousands of aphids thrive for several months or even for over a year. Here, in addition to colony defense against natural enemies, waste disposal is an important task for the gall dwellers to sustain their social life. In open galls, soldier nymphs actively clean colony wastes such as honeydew droplets, cast-off skins, and cadavers by pushing them with their head out of the gall opening. In the gall, the excreted honeydew is coated with aphid-derived powdery wax to form “honeydew balls,” which prevents the aphids from wetting and drowning with their own excretion. How the aphids deal with the accumulated honeydew in closed galls has been a mystery. Here, we report a novel gall-cleaning mechanism: the gall inner surface absorbs and removes the liquid waste through the plant vascular system. Such a plant-mediated water-absorbing property is commonly found in aphids forming closed galls, which must have evolved at least three times independently. By contrast, the inner surface of open galls is wax-coated and water-repelling, and in some cases, the inner surface is covered with dense trichomes, which further enhance the water repellency. In conclusion, gall-forming aphids induce novel plant phenotypes to manage the waste problems by manipulating plant morphogenesis and physiology for their own sake. This review describes our recent studies on waste management strategies by gall-forming social aphids and discusses future directions of this research topic.

## Introduction

**Figure 1:**
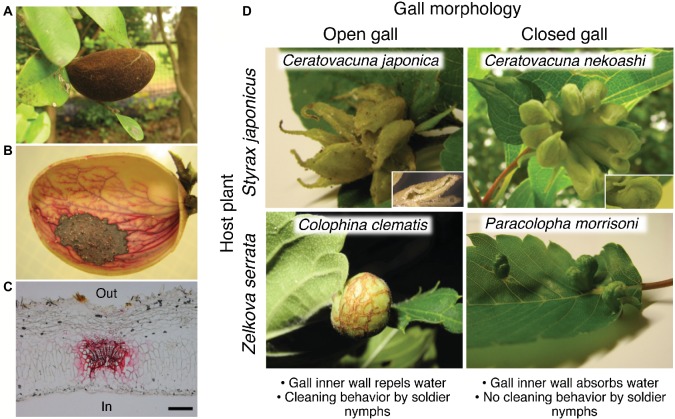
Open and closed galls of various aphids and water-absorbing property. **(A–C)** Closed galls of *N. monzeni* showing water-absorbing property. **(A)** A gall of *N. monzeni* on *D. racemosum*. **(B)** Safranin staining of the gall tissue, showing transported routes of absorbed water in red. **(C)** A histological section of the safranin-stained gall, in which the vascular bundle is conspicuously stained in red. Scale bar, 0.2 mm. **(D)** Waste management strategies in open and closed galls. Galls of *C. japonica* and *C. nekoashi* on *S. japonicus*, and *C. clematis* and *P. morrisoni* on *Z. serrata* are shown. A subgall of *C. japonica* with a slit opening and a subgall of *C. nekoashi* with no opening are also shown. Figures were modified from Kutsukake et al. (2012) and Uematsu et al. (2018). In, inner side of the gall; Out, outer side of the gall.

Aphids, exclusively living on plant phloem sap, embrace approximately 5,000 species in the world (Blackman and Eastop, 2000). Most of them form open colonies on their specific host plants, whereas no more than 10% of the aphids induce conspicuous galls on their host plants, whose morphology is quite characteristic and diverse (Figures 1A,D; Wool, 2005). Since the gall founder, called fundatrix or stem mother, forms a unique-shaped gall in a species-specific manner, the galling aphid species can usually be identified solely based on the gall morphology. This means that the morphological characteristics of the galls are mainly determined by aphid-derived genetic components rather than plant-derived ones, and for this reason such morphological traits of the galls are often regarded as “extended phenotypes” of the inducer insects (Stern, 1995; Inbar et al., 2004).

Most of the gall-forming aphids are restricted to the two subfamilies Eriosomatinae and Hormaphidinae in the family Aphididae (Wool, 2005). Their typical life history is complicated, where they have both sexual and parthenogenetic generations and alternate their host plants seasonally (Wool, 2005; Aoki and Kurosu, 2010). Briefly, a fundatrix appears from a fertilized egg in spring, induces a gall on the primary host plant, and produces offspring parthenogenetically in the gall, where the aphid colony experiences several parthenogenetic generations. Typically in early summer, winged adults appear and migrate to a different plant, namely the secondary host plant, where they also spend several generations parthenogenetically. Then, winged adults of a different type, called sexuparae, appear and return to the primary host plant to produce sexual females and males, where they mate and lay fertilized overwintering eggs that are to be fundatrices in next spring. Note that some hormaphidine species have multi-year life cycles, where they develop galls that last for over a year and thereby attain large colony sizes (Kurosu and Aoki, 2009; Aoki and Kurosu, 2010; Uematsu and Shibao, 2014). In addition to these morphs, many, if not all, gall-forming aphids are known to be social with altruistic morphs called “soldiers,” which are typically first- or second-instar nymphs specialized for colony defense (Stern and Foster, 1996; Abbot and Chapman, 2017). Soldier nymphs of some species also perform labors for nest maintenance including gall cleaning and gall repair (Aoki, 1980; Aoki and Kurosu, 1989; Kurosu and Aoki, 1991; Benton and Foster, 1992; Kurosu et al., 2003; Shibao and Fukatsu, 2003; Pike and Foster, 2004; Kutsukake et al., 2009, 2019; Lawson et al., 2014). Considering that all social species form galls at some point in their life cycle, gall formation is considered as one of the important ecological factors that have promoted social evolution in aphids (Aoki, 1987; Foster and Northcott, 1994; Stern and Foster, 1996; Pike and Foster, 2008).

For animals, especially those living in a nest, waste disposal is an essential issue to sustain a long-term survival. Aphid galls contain hundreds to thousands of insects and often continue for several months, or in some species even for over a year. Aphids suck plant phloem sap continuously and excrete plenty of sugar-rich honeydew. Accumulated honeydew within the gall would be fatal for inhabiting aphids due to contamination or drowning. How do the gall-forming aphids deal with the liquid wastes and sustain long-term social life? This review describes our recent findings of novel and unexpected biological solutions to the waste problems in aphid galls, in which aphids manipulate plant morphogenesis and physiology for their own sake to keep their social life healthy and safe. We also discuss future directions on this research topic.

## Gall Cleaning and Wax Production by Aphids in Open Galls

Gall morphology can be classified into two types, namely open galls and closed galls. The open galls possess opening(s) on the underside of the gall, so that aphids are able to dispose colony wastes through the openings. Previous studies reported that soldier nymphs in the open galls perform cleaning behavior by pushing or rolling honeydew balls, cast-off skins, and cadavers out of the openings (Aoki, 1980; Aoki and Kurosu, 1989; Benton and Foster, 1992; Uematsu et al., 2018). Inhibition of the waste disposal by turning the gall orientation upside down (the openings to be upward) resulted in high mortality of the aphids inside, indicating that gall cleaning is indispensable for survival of the gall inhabitants (Benton and Foster, 1992). Besides, aphids produce large amounts of powdery wax, which coats the excreted honeydew to form unsticky “marbles” or “honeydew balls” (Pike et al., 2002; Kutsukake et al., 2012; Uematsu et al., 2018). The wax-coated honeydew balls are repelled by the gall inner wall so that the aphids can easily push them without being wet or contaminated.

## Water Absorption and Honeydew Removal by Plant Tissues in Closed Galls

Waste disposal is impossible for aphids that form closed galls without openings. For a long time, it had been an enigma why the aphids living in completely closed galls can survive for a long period until the galls mature and finally form an exit for emigration. The answer to the mystery was presented in our research on a social aphid, *Nipponaphis monzeni*, that forms completely closed galls on the tree *Distylium racemosum* (Figure 1A; Kutsukake et al., 2012). *N. monzeni* is known to form an extremely long-lasting gall (taking some 2.5 years to maturity) that contains a large number of insects (over 2,000 aphids in mature galls) (Kurosu and Aoki, 2009). Despite the large colony size, we found no honeydew droplets accumulating within the galls, but only some powdery wax and cast-off skins. The possibility that aphids excreted little honeydew was rejected because, when the aphids were reared on an artificial feeding system (Shibao et al., 2002), we observed honeydew excretion. These observations led us to a hypothesis that the honeydew may be somehow removed from the inner cavity of the closed galls. In an attempt to verify the hypothesis, we injected 1 ml of food dye solution into natural galls of *N. monzeni* in the field and subsequently cut the galls to inspect the gall contents. Strikingly, no dye solution remained in the inner cavity of the galls 1 day after injection. Safranin solution injected into the gall cavity clearly stained the vascular bundles in red, indicating that the solution was absorbed by the plant tissues and removed through the vascular system (Figures 1B,C; Kutsukake et al., 2012). We also investigated whether the galls of *N. monzeni* are able to absorb sucrose solution, because aphid honeydew contains a large amount of sucrose. As the sucrose concentrations were elevated, the absorption efficiencies reduced: almost 100% absorption in 15 of 16 galls for 0% sucrose water; over 90% absorption in 6 of 10 galls for 2% sucrose water; 35–90% absorption in 8 of 10 galls for 4% sucrose water; and less than 40% absorption in all 11 galls for 8% sucrose water. Interestingly, the honeydew excreted by *N. monzeni* exhibited a low sugar content (less than 0.5% glucose), suggesting the possibility that the aphids may control their physiology to produce low-sugar honeydew that is easier for absorption by the gall inner surface. Considering that *N. monzeni* aphids produce plenty of powdery wax from their dorsal wax plates, we suggest the possibility that, although speculative, the aphids consume much sugar for massive production of the excreted wax, which might be relevant to the low sugar content in the honeydew of *N. monzeni*. As for possible mechanisms of water absorption of the plant side, we initially suspected two mechanisms, the passive water transportation driven by water potential of the plant tissue, and the active water transportation through water channels like aquaporins. From our data, the latter mechanism was unlikely because mercury chloride solution, an aquaporin inhibitor, did not affect the water absorption efficiency (Kutsukake et al., 2012). Plausibly, the passive water transportation mechanism driven by osmotic pressure-related water potential is involved in the water absorption property of the closed galls.

## Evolution of Water-Repelling/Absorption Properties in Aphid Galls

**Figure 2:**
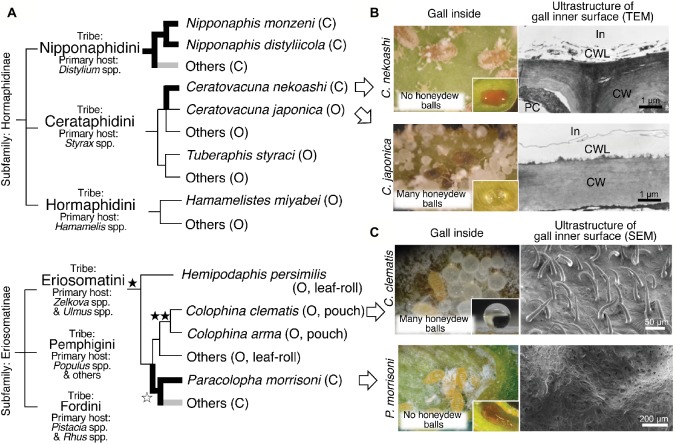
Evolution and plant surface structures of water-absorbing closed galls and water-repelling open galls. **(A)** A schematic phylogeny of the gall-forming social aphids and the evolution of water-absorbing/repelling properties. The occurrences of the water-absorbing closed galls are indicated in bold branches. The water-absorbing closed galls reported in Kutsukake et al. (2012) and Uematsu et al. (2018) are indicated in black, whereas others (potential water-absorbing galls) are indicated in gray. The evolution of dense trichomes on gall inner surface is indicated by black stars. High-density trichomes are indicated by a single star and very high-density trichomes are indicated by double stars. A white star indicates loss of trichomes. Closed (C) or open (O) galls are indicated in brackets. The phylogenetic relationship of gall-forming social aphids is based on Sano and Akimoto (2011) and Kutsukake et al. (2012). **(B)** Gall inside views and transmission electron micrographs of inner wall surface in galls of *C. nekoashi* and *C. japonica*. Hydrophobicity of the gall inner surface on which was placed a drop of food dye solution or water is also shown. **(C)** Gall inside views and scanning electron micrographs of the inner wall surface of galls of *C. clematis* and *P. morrisoni*. Note that aphid-derived wax was removed during the fixation procedure before the observation using a scanning electron microscope. Figures were modified from Kutsukake et al. (2012) and Uematsu et al. (2018). C, closed gall; CW, cell wall; CWL, cuticle wax layer; In, inner side of the gall; O, open gall; PC, plant cell.

Thus far, the water absorption property of closed galls was observed not only in *N. monzeni* but also in other aphid species (Figure 2A). In the subfamily Hormaphidinae, the water-absorbing closed galls were estimated to have evolved twice in the tribes Nipponaphidini and Cerataphidini independently. In the Nipponaphidini, *N. monzeni* and its allied species *Nipponaphis distyliicola* form water-absorbing closed galls (Kutsukake et al., 2012). Other allied nipponaphidine aphids also form closed galls in which no honeydew balls were detected, suggesting that their galls may absorb liquid wastes as well. In the Cerataphidini, *Ceratovacuna nekoashi* forms water-absorbing closed galls, whereas *Ceratovacuna japonica* and other Cerataphidini species form water-repelling open galls on *Styrax* trees (Kutsukake et al., 2012). In the subfamily Eriosomatinae, the water-absorbing closed galls have evolved at least once in the tribe Eriosomatini. *Paracolopha morrisoni* forms water-absorbing closed galls on *Zelkova serrata* leaves, whereas many other species, including *Colophina clematis*, form water-repelling open galls on the same *Zelkova* leaves (Uematsu et al., 2018). Taken together, water-absorbing property in the closed galls has evolved at least three times in the evolutionary history of the gall-forming aphids (Figure 2A).

## Manipulation of Structure and Hydrophobicity of Gall Inner Surface by Aphids

Here we focus on two congenic aphid species, *C. japonica* that forms water-repelling open galls and *C. nekoashi* that forms water-absorbing closed galls on the same tree *Styrax japonicus* (Figure 1D). *C. nekoashi* and *C. japonica* are both social species, whose life cycle and gall shape are quite similar. Their banana-bundle-shaped galls, that are transformed from axillary buds of a shoot by fundatrices, consist of approximately 10 subgalls with 50–100 insects per subgall (Kurosu and Aoki, 1988, 1990, 1994). Notably, however, *C. japonica* forms open galls wherein soldier nymphs actively clean wastes, whereas *C. nekoashi* forms closed galls wherein soldier nymphs do not clean (Kurosu and Aoki, 1988; Kurosu et al., 1990). Kutsukake et al. (2012) reported that no honeydew balls were found in the galls of *C. nekoashi* (Figure 2B), and food dye solution artificially introduced into the gall cavity was completely absorbed by the gall inner surface. The inner surface was hydrophilic on which the introduced dye solution rapidly spread (Figure 2B). By contrast, the gall inner surface of *C. japonica* was hydrophobic on which the introduced water was repelled and formed a sphere (Figure 2B). Ultrastructural observations revealed that the gall inner surface of *C. nekoashi* was covered with a reticular and spongy plant cuticle layer, whereas the gall inner surface of *C. japonica* was covered with a thick and dense cuticle layer (Figure 2B). These observations indicate that the cuticle wax structure determines the hydrophobicity and water-absorbing/repelling properties of the gall inner surface, which is determined by the galling aphids rather than by the host plant.

## Trichome Development and High Water Repellency in Some Open Galls

Recently, another intriguing phenomenon on insect-induced plant surface structure was discovered in galls of the wooly aphid *Colophina clematis*. This aphid forms pouch-shaped open galls on leaves of *Z. serrata* (Figure 1D). Uematsu et al. (2018) found that the gall inner surface was covered with a number of trichomes (Figure 2C), whose density was about 30 times higher than that on the non-galling area of the same leaf. The gall inner surface was covered with not only dense trichomes, but also aphid-derived hydrophobic wax particulates. Water droplets placed on the inner surface were highly repelled with contact angles of around 150°, whereas the water droplets on the wax-removed inner surface (trichomes only) were less repelled with contact angles of around 130°. The water droplets placed on normal non-galling leaf areas (with neither trichomes nor aphid wax) were not repelled with contact angles of less than 90°. Thus, the hydrophobicity of the gall inner surface of *C. clematis* was remarkably enhanced by the co-existence of the trichomes and aphid-derived hydrophobic wax, by which the aphids are able to clean the honeydew balls efficiently. Such microscale hierarchical structures on the organismal surface often contribute to water repellency of the surface, which is known as “lotus effect” observed on the surface of lotus leaves and other plant and animal surface (Barthlott and Neinhuis, 1997). Such massive trichomes were also found in pouch-shaped open galls of *Colophina arma*, an allied species of *C. clematis,* whereas no trichomes were detected in water-absorbing closed galls of *P. morrisoni* (Figures 1D,C, 2A). In leaf-rolling open galls of *Hemipodaphis persimilis*, the trichome densities were around a half of those in *C. clematis* galls (Uematsu et al., 2018). Thus, the trichomes developed in the open galls of the Eriosomatini species are regarded as another example of an extended phenotype of gall-forming social aphids.

## Conclusion and Future Directions

For waste management, gall-forming aphids employ either of the following strategies. In open galls, soldier nymphs dispose honeydew balls from gall openings, where the gall inner surface is hydrophobic, being covered with thick plant cuticle layer and sprinkled with aphid-derived powdery wax. In closed gall, liquid wastes are absorbed by hydrophilic gall inner surface, which is composed of plant-derived reticular and spongy layer, and removed through the vascular bundle system of the host plant. The two ecological traits, gall openness and waste removal strategies, seem to be tightly linked to each other among the gall-forming aphids, whereby colony defense and colony hygiene are harmoniously realized not only in open galls but also in closed galls. In a sense, water absorption in the closed galls can be regarded as “plant-mediated indirect social behavior” by the inducer aphids. While the water-absorbing closed galls have been identified in three gall-forming aphid lineages (Figure 2A), wider surveys of diverse gall-forming aphids (e.g., members of the tribes Pemphigini and Fordini) are needed to clarify the whole picture on this issue.

Another fascinating question on this research topic is molecular mechanisms underlying water-absorbing cuticle formation and water-repelling trichome development in the gall-forming aphids and the host plants. Upon gall formation, the fundatrix injects saliva into the plant tissue through the stylet (needle-like mouthpart). In this process, some bioactive molecules in the saliva may promote plant cell growth and cell division, hijack the plant developmental programs, and manipulate the plant morphologenesis and physiology for their own sake (Stone and Schönrogge, 2003; Raman et al., 2005). Such molecules may be effectors produced in the aphid salivary glands, although little is known about how insect effectors manipulate the plant morphogenesis and physiology to form the gall (Giron et al., 2016). In addition, phytohormones have been long believed to play a role in hypertrophy and hyperplausia of the plant cells in the gall tissues. Some studies detected high levels of phytohormones, such as auxin and cytokine, in the body or salivary glands of gall-forming insects, suggesting the involvement of phytohormones in the gall formation (Mapes and Davies, 2001; Tooker and De Moraes, 2011a,b; Yamaguchi et al., 2012). Hence, analyses of effectors and phytohormones in the salivary glands of the gall-forming aphids would be of great interest for further investigation. In addition, plant cuticle formation and trichome development have been well studied using the model plants, mainly *Arabidopsis thaliana* (Kunst and Samuels, 2009; Yeats and Rose, 2013; Pattanaik et al., 2014). In our study using non-model plants and insects, a candidate gene approach would be applicable, and the model plant researches will help unveil a molecular basis of the aphid-induced plant phenotypes described in this paper. Expectedly and hopefully, comparison between *C. japonica* vs. *C. nekoashi,* or *C. clematis* vs. *P. morrisoni*, which form open and closed galls on the same host plant, respectively, would unveil molecular components of the aphids and plants that are involved in the induction of water absorption/repellency of the galls.

## Author Contributions

MK wrote the manuscript. TF and KU critically revised the manuscript. All authors approved the final version of the manuscript.

### Conflict of Interest Statement

The authors declare that the research was conducted in the absence of any commercial or financial relationships that could be construed as a potential conflict of interest.
